# The business of deubiquitination – location, location, location

**DOI:** 10.12688/f1000research.7220.1

**Published:** 2016-02-11

**Authors:** Erin S. Coyne, Simon S. Wing

**Affiliations:** 1 Polypeptide Laboratory, Departments of Medicine and Biochemistry, McGill University, McGill University Health Centre Research Institute, Montreal, QC, Canada

**Keywords:** deubiquitination, deubiquitinating enzymes, DUB

## Abstract

A majority of proteins in the cell can be modified by ubiquitination, thereby altering their function or stability. This ubiquitination is controlled by both ubiquitinating and deubiquitinating enzymes (DUBs). The number of ubiquitin ligases exceeds that of DUBs by about eightfold, indicating that DUBs may have much broader substrate specificity. Despite this, DUBs have been shown to have quite specific physiological functions. This functional specificity is likely due to very precise regulation of activity arising from the sophisticated use of all mechanisms of enzyme regulation. In this commentary, we briefly review key features of DUBs with more emphasis on regulation. In particular, we focus on localization of the enzymes as a critical regulatory mechanism which when integrated with control of expression, substrate activation, allosteric regulation, and post-translational modifications results in precise spatial and temporal deubiquitination of proteins and therefore specific physiological functions. Identification of compounds that target the structural elements in DUBs that dictate localization may be a more promising approach to development of drugs with specificity of action than targeting the enzymatic activity, which for most DUBs is dependent on a thiol group that can react non-specifically with many compounds in large-scale screening.

## Introduction

The conjugation of the 76 amino acid peptide ubiquitin to proteins is an important post-translational modification that can modulate most if not all cellular processes. This occurs via the consecutive action of three enzymes: E1 ubiquitin-activating enzymes (two mammalian genes), E2 ubiquitin conjugating enzymes (~35 genes), and E3 ubiquitin ligases (~750 genes) ^1^. The E3 ligases are critical for conferring substrate specificity (reviewed in 2). Ubiquitin is covalently linked by its C-terminal glycine to, most commonly, the ε-amino group of a lysine on a target protein through an isopeptide bond. Occasionally, ubiquitin can be conjugated to cysteine, serine, threonine, and N-terminal methionine residues ^3– 7^. Subsequent ubiquitin moieties can be covalently linked to one of the seven lysine residues or the N-terminal methionine on the proximal ubiquitin to generate a polyubiquitin chain. Distinct functions are conferred depending on whether the protein is monoubiquitinated or polyubiquitinated and on the type of chain linkage. The two most common chain linkage types, K48 and K63, typically direct the substrate to different outcomes; the former is usually targeting proteins for degradation by the 26S proteasome, whereas the latter is generally involved in signal transduction, DNA repair, or endosomal sorting ^8– 10^. Conjugation of a linear chain of ubiquitins linked via their N-terminal methionines can serve to recruit proteins in cytokine signaling ^11, 12^. K11-linked chains ^13^ and probably the remaining chain types ^14^ can also target proteins to the proteasome. The 26S proteasome is a key mediator of intracellular protein homeostasis. It is an approximately 2.5-MDa macromolecular complex comprising a 20S cylindrical core particle capped at both ends by 19S regulatory particles. The 19S cap serves to recognize ubiquitinated substrates and allow their translocation into the lumen of the 20S core particle where the substrate then is hydrolyzed by the proteolytic machinery (reviewed in 2).

Importantly, ubiquitination is dynamic and reversible. Whereas early studies focused on understanding how ubiquitin is conjugated to substrates, recent years have seen markedly increased interest in deubiquitinating enzymes (DUBs). These studies have demonstrated many functions for deubiquitination, giving support to the notion that DUBs play equally important roles as ligases do in controlling ubiquitination.

A number of excellent reviews on DUBs have been published recently ^15– 18^. Therefore, in this commentary, we will highlight only the key concepts regarding the structure, functions, and mechanisms of these enzymes as these have been discussed in detail elsewhere. Instead, we will focus on the regulation of DUBs with emphasis on the role of localization in complexes and subcellular organelles in modulating their activities and function. We believe that such localization is a central factor in explaining how a relatively limited number of DUBs can exert a large range of specific functions. We will also comment briefly on the implications of this information on strategies for targeting DUBs for therapeutic purposes.

## Structure and mechanism

DUBs are peptidases that catalyze the cleavage of the bond formed between ubiquitin and substrate or ubiquitin and ubiquitin. There are approximately 90 DUBs in the human genome, compared with the more than 750 E3 ligases ^1^. There are five DUB families classified on the basis of the homology of their catalytic domains. These families are the ubiquitin C-terminal hydrolase (UCH), ubiquitin-specific protease (USP), ovarian tumor domain (OTU), Machado-Joseph disease (MJD), and Jab1/Mpn/Mov34 (JAMM) enzymes ( Table 1). All of these families are cysteine proteases, except for JAMM family members, which are metalloproteases. The crystal structures of members of each of these families have been solved (reviewed in 19). The catalytic mechanism of the cysteine protease DUBs is similar to that of plant papains, whereby cysteine, histidine, and aspartate residues form a catalytic triad where the histidine primes the cysteine for nucleophilic attack on the peptide bond and the aspartate aligns and polarizes the histidine. Metalloproteases require the co-ordination of a zinc ion for catalysis, which allows the abstraction of a hydrogen atom from a water molecule, generating a reactive hydroxyl ion capable of attacking the peptide bond.

**Table 1.** Families of deubiquitinating enzymes.

Family	Number	Deubiquitinating enzymes
Ubiquitin C-terminal hydrolase (UCH)	4	**UCHL1**, UCHL3, **UCHL5**, **BAP1**
Ubiquitin-specific protease (USP)	56	**USP1**, USP2, **USP3**, **USP4**, USP5, **USP6**, **USP7**, **USP8**, USP9X, USP9Y, USP10, USP11, **USP12**, USP13, **USP14**, **USP15**, **USP16**, USP18, **USP19**, **USP20**, **USP21**, **USP22**, USP24, **USP25**, **USP26**, USP27X, **USP28**, USP29, **USP30**, USP31, USP32, **USP33**, USP34, **USP35**, **USP36**, USP37, USP38, **USP39**, USP40, USP41, **USP42**, USP43, **USP44**, USP45, USP46, USP47, USP48, USP49, USP50, USP51, USP52, USP53, USP54, DUB3, **CYLD**, USPL1
Ovarian tumor (OTU)	16	OTUB1, OTUB2, OTUD1, OTUD3, OTUD4, OTUD5, OTUD6A, OTUD6B, OUT, YOD1, Otulin, **A20**, Cezanne, Cezanne2, TRABID, ACPIP1
Machado-Joseph disease (MJD)	4	Ataxin-3, Ataxin-3-like, JosD1, JosD2
JAB1/MPN/ Mov34 (JAMM)	11	PSMD7, **PSMD14**, EIF3H, **BRCC36**, CSCN5, CSCN6, **AMSH**, AMSH-LP, MPND, PRPF8, **MYSM1**

Enzymes cited in this commentary are shown in bold.

## Function

Maintaining an adequate pool of free ubiquitin available for immediate conjugation is essential for the ability of the ubiquitin system to respond rapidly to changing cellular needs. DUBs play several critical roles in this general function of maintaining free ubiquitin. Ubiquitin is encoded in the human genome as four distinct genes: the two polyubiquitin genes *UBB* and *UBC* and the ubiquitin-fusion genes *UBA52* and *RPS27A*, which encode a single ubiquitin protein fused to the ribosomal proteins L40 and S27A, respectively. Thus, ubiquitin is synthesized *de novo* as fusion proteins that must be cleaved to generate free ubiquitin by DUBs. The free ubiquitin pool is also maintained by recycling ubiquitin that has been released from proteins just prior to destruction by either the proteasome or the endocytic, lysosomal pathway. DUBs act at both locations to provide this recycling function (see below). Finally, DUBs also contribute ubiquitin to the free pool through their removal of ubiquitin from specific substrates with the effect of reversing or preventing the effects of ubiquitination. DUBs can also act to edit or remodel ubiquitin chains on substrates ^20^ and thus may redirect their fate ^21, 22^. The extent to which DUBs play such a remodeling role remains unknown. But the identification of DUBs which act on specific chain linkages—e.g. AMSH ^23^ and Ataxin3 ^20^ for Lys 63-linked ubiquitin—clearly makes such a function plausible. Interestingly, DUBs can also inhibit conjugation by binding to the E2 and interfering with ubiquitin transfer to the E3 ^24, 25^.

## Regulation

The many fewer DUBs compared with ubiquitin ligases suggest that DUBs may have much broader specificity with many more substrates per DUB than per ligase. Therefore, regulation of their activity is critical to maintain specificity and occurs through both intramolecular and external factors (reviewed in Sahtoe and Sixma ^18^). Indeed, evidence to date indicates that DUBs employ all the classic mechanisms of enzyme regulation in sophisticated fashions.

*Regulation of expression* is well documented. Variation of expression of some enzymes in tissue(s)/cell type ^15^ or upon specific stimuli ^26^ represents one layer of control which can allow DUBs to have specific effects. Such regulation of expression takes place through both transcriptional and post-transcriptional mechanisms, including regulation by miRNAs ^27^. Furthermore, regulation of enzyme levels by cleavage or degradation also occurs. USP1 cleaves itself following ultraviolet irradiation, leading to accumulation of the DNA replication processivity factor PCNA ^28^. The OTU domain containing A20 can be inactivated by cleavage by MALT1, a protein associated with mucosa-associated lymphoid tissue lymphoma ^29^. A number of DUBs exist in complex with E3s. The DUBs often can modulate ubiquitination of the E3 ^30– 32^ as well as ubiquitination by the E3—whether of itself or other substrates (e.g. USP7 on p53 ^33^ and its ligase Mdm2 ^34^)—but the E3 can also modulate the stability of the DUB ^35^.

*Substrate activation* – The apo enzymes are often in an inactive state and this is due to being in a conformation that does not allow catalysis or due to auto-inhibitory loops that impair substrate access to the active site. Binding of the substrate ^36^ or the ubiquitin portion of the substrate ^37^ can result in reorganization of the enzyme into a conformationally active form, indicating that substrate activation is an important regulatory mechanism.

Regulation by *post-translational modification* is also well described with examples of modulation of activity by phosphorylation ^38, 39^, sumoylation ^40^, and ubiquitination ^41, 42^. Furthermore, recent studies indicate that reactive oxygen species can inactivate many DUBs in a reversible manner by oxidizing the active-site cysteine to a cysteine sulphenic acid or sulphene amide ^43– 45^.

*Allosteric regulation* due to binding of other proteins to the DUB is well described with examples of both activation ^46, 47^ and inhibition ^48, 49^ of enzyme activity.

*Localization of the enzyme* is becoming an increasingly appreciated regulatory mechanism allowing action on substrates that might not otherwise occur at significant rates if both enzyme and substrate were freely circulating, diluted in the cytoplasm. Here, we will highlight a few well-developed examples of localization – either to intracellular complexes or to organelles ( Table 2) as a regulatory layer for DUB function. In many cases, the localization to a complex also results in allosteric regulation of the enzyme. A systematic study of localization of GFP-tagged DUBs indicates that approximately 25% of the enzymes are found in specific subcellular structures ^50^.

**Table 2.** Subcellular localization of some deubiquitinating enzymes.

Cellular compartment	Family	Deubiquitinating enzymes	Reference
Nucleus	USP	USP1	81
		USP3	78
		USP4	82
		USP7	83
		USP16	77
		USP21	84
		USP22	85
		USP26	86
		USP28	87
		USP36	88
		USP39	89
		USP44	90
	UCH	UCHL5	91
		BAP1	92
	MJD	ATXN3	93
	JAMM	BRCC3	76
		MYSM1	75
Mitochondria	USP	USP8	65
		USP15	64
		USP15
		USP30	61
		USP35	62
Endoplasmic reticulum	USP	USP19	71
		USP20	94
		USP25	95
		USP33	94
	UCH	UCHL1	96
Golgi	USP	USP32	97
		USP33	98
Endosome	USP	USP8	53
	JAMM	AMSH	52

JAMM, JAB1/MPN/Mov34; MJD, Machado-Joseph disease; UCH, ubiquitin C-terminal hydrolase; USP, ubiquitin-specific protease.

### PMSD14, UCHL5, USP14, and the proteasome

The DUBs PMSD14/Rpn11, UCHL5/UCH37, and USP14 are all associated with the 19S regulatory cap of the proteasome; PMSD14 is a constituent component, whereas UCHL5 and USP14 are reversibly associated proteins. Upon binding to the 19S cap, UCHL5 and USP14 undergo restructuring, resulting in greatly increased enzymatic activity. Binding of UCHL5 to the proteasome repositions a crossover loop, thereby relieving an auto-inhibitory effect and allowing substrate access to the active site ^48^. Similarly, in USP14, the ubiquitin-binding pocket is obscured by two loops and binding to the 19S regulatory cap reveals the ubiquitin-binding site necessary for deubiquitination ^51^. The localization of these DUBs gives them specific access to substrates associated with the proteasome. PMSD14 cleaves the ubiquitin chain at its junction with the substrate, thereby allowing efficient unwinding and insertion of the substrate into the 20S core and so its DUB activity promotes proteolysis. Both USP14 and UCHL5 have been shown to deubiquitinate and impair proteolysis of some substrates. However, it remains possible that, for some substrates, these enzymes may edit chains into forms which allow more effective binding to or processing by the proteasome.

### AMSH and USP8 and ESCRT complexes

Receptor endocytosis followed by either recycling to the plasma membrane or trafficking through multi-vesicular bodies (MVBs) to lysosomes for degradation plays an important role in modulating signal transduction. During endocytosis, monoubiquitination and polyubiquitination of the receptor constitute a sorting signal that can be decoded by the ESCRT (endosomal sorting complex required for transport) complexes. The four ESCRT complexes (ESCRT-0, -I, -II, and -III) function together to generate MVBs by allowing the remodeling of the plasma membrane and the budding and internalization of cargo-bearing vesicles. The contents of MVBs then are sent to the lysosome for degradation. Two DUBs are known to associate with ESCRT complexes in the endocytic/lysosomal pathway. AMSH (a JAMM family member with specificity for K63 chains) and USP8 associate with STAM proteins, a component of the ESCRT-0 complex ^52, 53^. AMSH and USP8 can also interact with CHMP proteins that are components of the late ESCRT-III machinery ^23, 54^. Association with ESCRT is necessary for AMSH function in endocytosis ^55^ and disruption of this association causes accumulation of the EGF receptor via impaired degradation ^56^. Thus, AMSH activity enhances receptor trafficking toward lysosomal degradation. USP8 can be both a positive and a negative regulator of receptor endocytosis. Loss of USP8 leads to hyperubiquitination and enhanced degradation of EGF, MET, and ERBB3 receptors ^57, 58^ but increases the level of the Wnt receptor Frizzled by enhancing receptor recycling ^59^. The contrasting effects of loss of AMSH and USP8 on receptor stability as well as their differences in chain linkage specificity indicate that these ESCRT-associated DUBs have distinct functions.

### USP30 and the mitochondria

Mitochondrial dysfunction can have profound effects on cell function and viability. Indeed, mitochondrial dysfunction and impaired clearance of damaged mitochondria are hallmarks of the neurodegenerative disorder Parkinson’s disease. The ubiquitin ligase Parkin is mutated in an autosomal recessive form of the disease. In Parkin-mediated mitochondrial clearance (mitophagy), the kinase PINK1 accumulates on damaged mitochondria and recruits Parkin to ubiquitinate a variety of substrates on the mitochondria (reviewed in 60). USP30 was first identified as a DUB with a mitochondrial targeting sequence that is embedded in the mitochondrial outer membrane and plays a role in mitochondrial dynamics ^61^. Subsequent studies revealed that it is a negative regulator of mitophagy. USP30 antagonizes Parkin-mediated mitophagy by deubiquitinating its target substrates ^62^. It has also been shown to delay the recruitment of Parkin to damaged mitochondria ^63^. Other DUBs may also co-localize to the mitochondria and play a role in mitophagy. USP15 antagonizes Parkin mitochondrial ubiquitination ^64^, USP35 can delay Parkin-mediated mitophagy through unclear mechanisms ^63^, and USP8 removes K6-linked polyubiquitin chains from Parkin itself to facilitate its recruitment to damaged mitochondria ^65^. Additionally, ubiquitin itself can be phosphorylated on serine 65 by PINK1 ^66– 68^. Interestingly, mitochondrial DUBs, including USP30, USP8, and USP15, are impaired at hydrolyzing these phosphoUb chains ^69^, thus providing an additional regulatory mechanism for Parkin-mediated mitophagy.

### USP19 and the endoplasmic reticulum

USP19 was first identified as a DUB upregulated in skeletal muscle during catabolic conditions ^70^. It is expressed as two major isoforms: one cytoplasmic and the other with a transmembrane domain that results in anchoring of the C-terminal tail of the protein in the endoplasmic reticulum (ER) membrane with retention of the catalytic domain in the cytoplasm ^71^. Overexpression of USP19 has been shown to rescue model substrates from ER-associated degradation (ERAD) ^71^ as well as an ER-localized ligase MARCH6 ^72^. However, silencing of USP19 does not affect levels of ERAD substrates in a consistent manner ^72, 73^, so its physiological functions at the ER remain unclear. USP19 can inhibit myogenic differentiation through suppression of an unfolded protein response that is required for muscle cell fusion ^74^. Interestingly, these effects are dependent on catalytic activity and occur with the ER but not the cytoplasmic isoform although both isoforms’ catalytic domains are in the cytoplasm, indicating that the localization is critical for its ability to deubiquitinate specific substrates ^74^.

### Chromatin deubiquitinating enzymes

Histone modifications are critical for DNA-dependent processes, including repair, replication, and transcription. Many DUBs have been shown to remove ubiquitin from chromatin, most commonly from histones H2A and H2B. MYSM1 and BRCC36 are two JAMM family members known to deubiquitinate H2A, with BRCC36 preferentially removing K63 polyubiquitin and suppressing DNA repair pathways. Importantly, MYSM1 and BRCC36 are associated with complexes that activate their deubiquitinating activity. MYSM1 is active as part of the 2A-DUB complex ^75^, whereas BRCC36 is associated with the BRCA1-A complex and this association activates its activity in the nucleus ^76^. Other DUBs capable of deubiquitinating histones include USP3, USP7, USP12, USP16, USP21, USP22, and USP44. Disruption of some of these DUBs results in altered cell cycle progression. Although USP3 and USP16 are not known to associate with any complexes, depletion of either enzyme results in aberrant cell cycle progression, with USP16 knockdown resulting in impaired mitosis ^77^ and depletion of USP3 resulting in a delay in S-phase progression ^78^. These different outcomes suggest that localization of DUBs to specific chromatin loci results in differential gene expression. DUBs could also target non-histone substrates at these sites to contribute to the phenotype.

## Pharmacological targeting

Given the role of ubiquitination in many important processes that are deranged in disease, it is an attractive set of enzymes for pharmacological intervention. Targeting deubiquitination is also alluring in that the more limited number of DUBs in comparison with ligases makes phenotypic screens of DUBs more feasible. Indeed, the availability of small hairpin RNA (shRNA) or small interfering RNA (siRNA) libraries targeting all of the DUBs allows relatively rapid determination of whether loss of function of a DUB will yield a desirable phenotype. The limited number of DUBs allows screening to be applied to even relatively low-throughput assays.

Once loss of function of a DUB is shown to produce an effect that might be clinically desirable, the development of that observation into a potential drug is much more challenging. Although a number of assays of DUB activity are available and amenable to high-throughput screens of large compound libraries and have resulted in some lead compounds (reviewed in 79), these assays are at high risk of yielding many unproductive hits as almost all of the DUBs are thiol-based proteases and the highly reactive thiol group of the catalytic cysteine residue is well recognized to react non-specifically to many compounds. Inhibition of the enzymatic activity may not be desirable for other reasons. The large number of potential substrates for each DUB may lead to many more undesirable effects arising from inhibition of catalysis. Whole body gene inactivation of the enzyme in mice may be helpful in predicting the extent of such adverse effects.

A number of alternative strategies can be proposed to inhibit DUBs; however, these strategies require significantly more characterization of the enzymes. Since the enzymes must bind ubiquitin, an alternative approach to broad inhibition of a particular enzyme would be to inhibit its ability to bind ubiquitin. Generally, such inhibition would require knowledge of the structure of the enzyme bound to ubiquitin to identify the essential elements of the ubiquitin-binding domain(s). A recent mutagenic strategy has created ubiquitin variants that are able to bind and inhibit enzymatic activity ^80^. Remarkably, variants that were selective for specific DUBs were obtained. Crystallization of the ubiquitin variant/DUB complex identified specific residues on the DUB that contact the variant and that yield specificity for a particular enzyme. Some DUBs have multiple ubiquitin-binding sites that permit binding of ubiquitin chains. These likely restrict the orientation of the ubiquitins in the chain that can be accommodated and therefore result in specificity of the enzyme for particular chain cleavages. Thus, inhibition of one of these specific domains may result in interference with ubiquitin chain binding or with the chain linkage specificity of the enzyme.

Targeting specific functions of a DUB may be achieved by identification of the specific substrates that mediate these effects. Subsequent structure function analyses then can identify the interacting domains of the enzyme and substrate and lead to the development of assays that can screen for compounds that interfere with the interaction. As discussed earlier, an important determining factor for both substrate specificity and regulation is the localization of the DUB. Once the mechanisms that result in targeting to a specific compartment or complex are determined, then assays that measure this binding can be similarly designed to screen for inhibitors. Since there are several examples in which complex formation or substrate binding activates the DUB, inhibitors that stabilize the enzyme in the auto-inhibited form could be developed. All of these approaches will rely heavily on structural studies both to help design the assay and to confirm that the identified compounds are functioning through the expected mechanisms.

## Closing perspectives

Much progress has been made in our understanding of DUBs over the past 15 years. Structures have now been solved for many DUBs. However, in a number of cases, only the catalytic domain has been resolved and it is clear that important information resides in the other regions of the enzyme and will be needed to improve success in pharmacological targeting. A much larger gap in our understanding is in the functions of the enzymes, both at a molecular level (substrate identification) and at a cellular or whole organismal level (the physiological effects of removal of ubiquitin from the substrates). The former may be addressed through analysis of differential ubiquitination of proteins upon loss of function of the DUB. Although much progress has been made in ubiquitinome analysis, it remains to be determined whether current methods are sufficiently reproducible and precise to detect what might be small differences in steady-state levels of ubiquitination that arise upon DUB inactivation. The availability and application of methods for RNA silencing, gene editing, and gene knockout have been transformative in permitting the elucidation of physiological functions. However, such information remains available for only a small minority of the DUBs. Finally, our understanding of regulation of DUBs has progressed significantly. All of the classic types of enzyme regulation are present, but localization within organelles and complexes, allosteric regulation within complexes, and substrate activation appear to be prominent mechanisms which together allow tightly regulated activity on specific substrates. Identifying the structural elements underlying these mechanisms will offer the potential of targeting them to obtain drugs with highly specific effects.
